# Proactive Information Sampling in Value-Based Decision-Making: Deciding When and Where to Saccade

**DOI:** 10.3389/fnhum.2019.00035

**Published:** 2019-02-11

**Authors:** Mingyu Song, Xingyu Wang, Hang Zhang, Jian Li

**Affiliations:** ^1^School of Psychological and Cognitive Sciences and Beijing Key Laboratory of Behavior and Mental Health, Peking University, Beijing, China; ^2^Princeton Neuroscience Institute, Princeton University, Princeton, NJ, United States; ^3^Department of Industrial Engineering and Management Sciences, Northwestern University, Evanston, IL, United States; ^4^PKU-IDG/McGovern Institute for Brain Research, Peking University, Beijing, China; ^5^Peking-Tsinghua Center for Life Sciences, Beijing, China

**Keywords:** decision-making, eye-tracking, information sampling, Bayesian inference, drift-diffusion model

## Abstract

Evidence accumulation has been the core component in recent development of perceptual and value-based decision-making theories. Most studies have focused on the evaluation of evidence between alternative options. What remains largely unknown is the process that prepares evidence: how may the decision-maker sample different sources of information sequentially, if they can only sample one source at a time? Here we propose a theoretical framework in prescribing how different sources of information should be sampled to facilitate the decision process: beliefs for different noisy sources are updated in a Bayesian manner and participants can proactively allocate resource for sampling (i.e., saccades) among different sources to maximize the information gain in such process. We show that our framework can account for human participants' actual choice and saccade behavior in a two-alternative value-based decision-making task. Moreover, our framework makes novel predictions about the empirical eye movement patterns.

## Introduction

Value-based binary choice is a common and fundamental form of human decision making, from choosing between ham and turkey sandwiches for lunch to determining whether to have a family with a particular individual. During these decisions, the process of evaluating the options and comparing them is often complex: even in problems as simple as deciding on which sandwich to take, people usually need to gaze at different options sequentially for multiple times before arriving at a decision.

Classic theories about evaluation often neglect gazing and fixation, merely focusing on how values of individual items are assigned (Kahneman and Tversky, 1979; Levy and Glimcher, 2012; Ruff and Fehr, 2014). Recent studies have started to pay attention to the important role of fixation in binary and multiple choice scenarios and have typically viewed fixation as an evidence accumulation process (Krajbich et al., 2010; Krajbich and Rangel, 2011; Cassey et al., 2013; Towal et al., 2013; Tavares et al., 2017). Recent primate neurophysiology and human neuroimaging research has placed this accumulation process at the core for perceptual decision making. It has been hypothesized that noisy evidence for each decision accumulates until certain threshold is reached and the corresponding decision is made (Ratcliff, 1978; Shadlen et al., 1996; Platt and Glimcher, 1999; Gold and Shadlen, 2002; Bogacz et al., 2006; Summerfield and Tsetsos, 2012; McGinty et al., 2016). Such a computational approach has also been adopted to study the process of value-based decisions (Krajbich et al., 2010; Krajbich and Rangel, 2011; De Martino et al., 2012). In one such study (Krajbich et al., 2010), human participants were asked to choose between two snack items on a computer screen. Participants could look at both items freely before making the choice and their eye movement data were simultaneously recorded. In most trials, participants' fixation switched back and forth between the two items for a few times before the final choice was made. By assuming that the fixated and non-fixated items were sampled asymmetrically and adopting an attentional drift-diffusion model (aDDM), Krajbich et al. successfully predicted participants' choices based on the observed eye tracking data. As in other previous studies, they concentrated on how evidence is integrated to reach the decision threshold given the fixation pattern shown by the participants, and aDDM is just one form of the stochastic accumulation models that also include sequential probability ratio test (Gold and Shadlen, 2002, 2007), race and leaky competing accumulator models (Usher and McClelland, 2001), among others (Bogacz et al., 2006). In most of previous studies, fixation data were taken as given and experimentally measured saccade data (via eye-tracking) were fed into the models to predict choice behavior (Krajbich et al., 2010; Krajbich and Rangel, 2011; but see Towal et al., 2013). Here we focus instead on the sampling assumption itself: What drives the switching of fixation between options in a two-alternative value-based choice task before the choice is made?

In the current study, we propose a Bayesian proactive sampling framework to account for both the choice behavior and saccade patterns in the same experiment run by Krajbich et al. (2010). We assume that instead of a single quantity, item attractiveness is internally represented as a probability distribution along the value dimension, and the fixation duration reflects the number of samples gleaned from such underlying distribution to form a belief distribution (Cassey et al., 2013). In this way, we formulate the evaluation process as Bayesian belief updating based on samples from different information sources rather than simple evidence accumulation (Cassey et al., 2013). More importantly, inspired by the Informax algorithm (Butko and Movellan, 2010), we assume that participants proactively switch their fixation from one item to the other when the marginal information gain of continuing the current fixation becomes lower than that of switching. For instance, fixating at one item (and gathering information/samples from it) for too long might not be beneficial, since the participant would have been very confident about how attractive the fixated item is but still uncertain about its alternative, rendering inability to choose between the two items. Thus, to make efficient decisions, participants need to balance between getting a more accurate estimation on the currently fixated item by continuously sampling and potentially more information gain by switching fixation to the other item. Similar ideas of active sampling have also been proposed in the field of visual search and in perceptual decision tasks (Najemnik and Geisler, 2005; Cassey et al., 2013; Ahmad et al., 2014).

Similar to aDDM (Krajbich et al., 2010) and the value-plus-salience model (Towal et al., 2013), our model well predicts participants' choice behaviors: for example, the decisions bias toward the last fixated item and the item fixated longer. Furthermore, our model predicts the distribution of fixation durations. It does so from a Bayesian perspective and can explain fixation patterns that previous stochastic accumulation models such as aDDM were agnostic about: for instance, the fixation duration is shorter in trials with greater absolute rating difference between items and for later fixations within a trial. Most importantly, our model views the saccade switching phenomena as an active process to maximize information gain in order to reach a decision more efficiently. Our approach thus provides a unified framework in describing how different sources of information are sampled proactively to facilitate the decision process.

## Materials and Methods

### Task

The experimental design and data collection were reported in detail in Krajbich et al. (2010). In brief, 39 Caltech students participated in the experiment and they were asked to refrain from eating 3 h before the task. The experiment consisted of a rating phase and a choice phase.

In the rating phase, participants were asked to rate 70 different food items using an on-screen slider bar (“how much would you like to eat this at the end of the experiment?”), on a scale of −10 to 10. Any item receiving a rating lower than 0 would not show up in the following choice phase so that all choice items are motivationally relevant to the participant.

**Figure 1:**
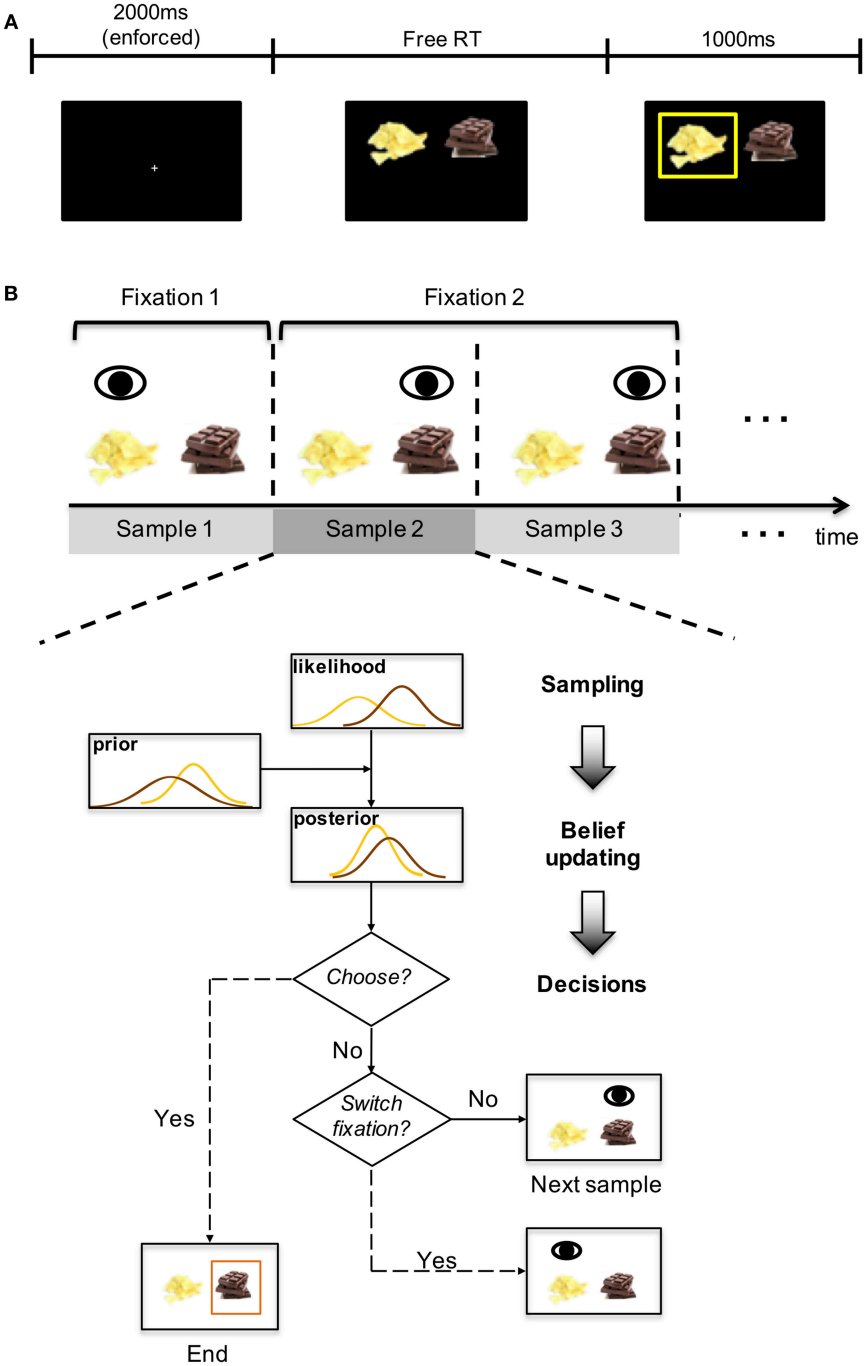
Experiment design and diagram of the model. **(A)** experiment design. In each choice trial, participants were presented with images of two food items and asked to make their choices. After the choice was made, a yellow box appeared around the chosen item for 1 s. See Figure 1 of Krajbich et al. (2010). **(B)** the upper panel illustrates the fixation sequence. Each fixation consists of at least one sample. The lower panel shows the four stages of the model: sampling, belief updating, decision and fixation switch. The yellow and brown curves correspond to the two items.

In each trial of the choice phase, participants were asked to choose from a pair of food items (selected from the 70 items they rated earlier) by pressing the left or right key on the keyboard (Figure 1A) while their eye movements were simultaneously recorded by the eye-tracker. The spatial locations of these snack items were randomized across trials. There was no time limit for response. In the end, participants were paid $20 show-up fee in addition to the snack item they picked in a random trial of the choice phase. For details on the choice phase we refer the readers to the original paper (Krajbich et al., 2010).

### Model

We propose a sampling-and-inference based model (Figure 1B) to predict both the choice and eye movement patterns leading to the decision. Instead of viewing gaze switches between options merely as an evidence accumulation process, we reason that this process is carried out to maximize the informational gain to differentiate between two estimated value distributions. In this section, we first briefly lay out the structure of the model, and then describe the assumptions and predictions in detail.

On each trial, we assume that the participant goes through a few gaze switch cycles, each of which contains information-collection and decision-making steps. The participant's goal is to make the correct choice (i.e., the item with greater attractiveness) as quickly as possible. Due to the span of attention, information collection is inevitably biased toward the fixated item and gaze switch is a natural means to maximize the information gain. More concretely, we hypothesize that during the information-collection cycle, the participant chronically (a) samples noisy evidence from the two items, and (b) updates their internal beliefs about the values of the two items accordingly. During the decision-making step, the participant (c) judges whether the information collected is enough to warrant a decision, i.e., the decision variable surpassing a threshold; and if so, a decision is made. Otherwise, the participant (d) chooses which item to fixate on next contingent on the relative information gain between the two items.

### (a) Sampling (With Bias)

In the beginning of each trial, the participant randomly decides which item to look at [with 74% probability of looking at the left item first and 26% of the right, based on the empirical fixation probability (Krajbich et al., 2010)]. At any specific moment, the two items are referred to as the fixated item (denoted by *f*) and the non-fixated item (denoted by *n*). We assume that the participant has no direct access to the true value of either item (*v*_*f*_ and *v*_*n*_) but can only obtain random samples from a Gaussian distribution centered around the true value (*t* denotes the *t*-th sample):

(1) $$\begin{array}{r} x_{f,t}\sim N\left(v_{f},\sigma_{f}^{2}\right) \end{array}$$

(2) $$\begin{array}{r} x_{n,t}\sim N\left(\gamma v_{n},\sigma_{n}^{2}\right) \end{array}$$

For the fixated item (see Equation 1), the mean of the sampling distribution is set to be the participant's rating of that item in the rating phase, under the assumption that their rating upon contemplation for each item reflects an accurate and unbiased estimation of the true item value. The variance of sampling distribution is denoted by $\sigma_{f}^{2}$ ($\sigma_{f}^{2}=\sigma_{0}^{2}$, with σ_0_ being a free parameter of the model). Similar to Krajbich et al. (2010), we assume that the non-fixated item is perceived with distortion. For simplicity, we scale the mean and variance of its sampling distribution by factors γ (0 ≤ γ ≤ 1) and κ ($\left(\sigma_{n}^{2}=\kappa \sigma_{0}^{2};\kappa \geq 1\right)$ respectively to reflect the discounted and noisier representation for the non-fixated item.

Sampling takes time and we simply assume the sampling time follows a uniform distribution between 50 and 150 ms, based on the empirical observations in object recognition (Kirchner and Thorpe, 2006) and visual working memory studies (Gegenfurtner and Sperling, 1993) that it takes about 100 ms for visual information to be extracted or transferred from iconic memory to visual working memory.

The samples *x*_*f, t*_ and *x*_*n, t*_ will then be used to update the participant's belief of the values of corresponding items.

### (b) Updating

We formulate the belief updating procedure according to the Bayes' rule. First, starting from the internal representation of item values, we assume that the participant has a broad prior over the values of two items at the beginning of each trial, centered around zero with a variance of $\sigma_{i,0}^{2}=\sigma_{0}^{2}$, where *i* = *f* or *n* (fixated or non-fixated).

With the samples obtained from both items, the participant updates their beliefs about item values by combining the likelihoods of samples (*x*_*f, t*_ and *x*_*n, t*_) and prior beliefs to form the posterior beliefs according to the Bayes' rule:

(3) $$\begin{array}{r} P\left({\hat{v}}_{i}=v|x_{i,1:t},\sigma_{i}^{2}\right)\propto P\left(x_{i,t}|{\hat{v}}_{i}=v,\sigma_{i}^{2}\right)P\left({\hat{v}}_{i}=v|x_{i,1:t-1},\sigma_{i}^{2}\right) \end{array}$$

where ${\hat{v}}_{i}$ (*i* = *f* or *n*) is the value estimate. Since both the prior and likelihood are assumed to be Gaussian, the participant's posterior beliefs are also Gaussian (Lee, 2012) (denoted by $N(\mu_{i,t},\sigma_{i,t}^{2}))$), with their means updated according to

(4) $$\begin{array}{r} \mu_{i,t}=\frac{\sigma_{i,t-1}^{2}x_{i,t}+\sigma_{i}^{2}\mu_{i,t-1}}{\sigma_{i,t-1}^{2}+\sigma_{i}^{2}} \end{array}$$

after the *t*th samples.

The variance of the posterior belief about the fixated item is

(5) $$\begin{array}{r} \sigma_{f,t}^{2}=\frac{\sigma_{f,t-1}^{2}\sigma_{f}^{2}}{\sigma_{f,t-1}^{2}+\sigma_{f}^{2}} \end{array}$$

For the non-fixated item, we assume a variance expansion effect (Bogacz et al., 2007; Bornstein et al., 2017). In particular, we hypothesize that the participant becomes more uncertain about the non-fixated item while they are fixating the other item so that the variance of the posterior belief about the non-fixated item is the same as Equation 5 except that an extra expanding factor λ (>1) is introduced:

(6) $$\begin{array}{r} \sigma_{n,t}^{2}=\lambda \frac{\sigma_{n,t-1}^{2}\sigma_{n}^{2}}{\sigma_{n,t-1}^{2}+\sigma_{n}^{2}} \end{array}$$

### (c) Judging Whether Information Is Enough for a Decision

Similar to the aDDM model in previous research (Krajbich et al., 2010; Tavares et al., 2017), we use the relative decision value ($RDV=|{\hat{v}}_{f}-{\hat{v}}_{n}|$) as the decision variable. At the beginning of each trial, the RDV starts at 0 and with the belief updating after each sample, the participant continuously evaluates the probability of making a correct choice, according to their value estimates for the two items. That is, $P\left({\hat{v}}_{f}-{\hat{v}}_{n}>0\right)$ if ${\hat{v}}_{f,t}>{\hat{v}}_{n,t}$ and $P\left({\hat{v}}_{n}-{\hat{v}}_{f}>0\right)$ otherwise. If this probability exceeds a threshold θ_*t*_, the participant will pick the item with the higher estimated value, and the sampling-and-decision procedure terminates. Otherwise, the participant continues to collect more information until such a fair comparison is warranted. We assume that the threshold θ_*t*_ decreases after each belief update, in order to avoid arbitrarily long arbitration (Tajima et al., 2016). For simplicity and without loss of generality, we use a linear function: θ_*t*_ = 1−δ*t*, where again *t* denotes the number of samples or updates.

### (d) Choosing Which Item to Fixate On

Here we assume that if the threshold for choice decision has not been reached, the participant decides whether to switch fixation in such a way as to separate two value distributions most efficiently. Inspired by the optimal sampling theory in perceptual decision making that the sampling time allocated to different information sources should be proportional to their noise levels (Cassey et al., 2013), we assume the probability of switching to the non-fixated item is determined by a logistic function of the uncertainty (standard deviation) ratio between the posterior belief distributions:

(7) $$\begin{array}{r} P_{Switch,\;t}=\frac{1}{1+e^{-\left(\omega \frac{\sigma_{n,t}}{\sigma_{f,t}}+\omega_{0}\right)}} \end{array}$$

where ω (> 0) reflects the sensitivity to the uncertainty ratio, and ω_0_ reflects a bias on saccade (“repositioning”) tendency respectively. Note that the fixated item becomes non-fixated and vice versa (corresponding to a swap of subscripts *f* and *n* in the equations) once the saccade switch occurs.

This saccade policy arises from our assumption that prior to a final decision, the participant actively samples from the two items so that they can reach a decision efficiently. Intuitively, if the non-fixated item bears a much higher uncertainty relative to the currently fixated one, the participant should switch fixation to the non-fixated item to gain more information. The proactive sampling continues until the decision threshold has been reached and an explicit decision ensues.

### Comparison With aDDM

As pointed out in previous literatures, Bayesian update with sampling from Gaussian distributions (assuming equivalent sampling variance for the fixated and non-fixated items and no expansion of variance for the non-fixated item) is essentially equivalent to the combination of evidence-accumulation and Wiener process in aDDM (Bitzer et al., 2014). The main difference between our work and previous studies, however, is that we proposed a fixation-switch policy based on active sampling theory, which predicts the patterns of both fixations and the final choice, whereas Krajbich et al. (2010) used the empirical fixation patterns to derive the choice pattern.

### Simulation

Given the multiple dimensions of fixation data (the number of fixations, the fixation duration, and other fixation patterns) and choice behavior, it is therefore difficult to devise a single metric to perform model fitting. Instead, we perform model simulation under a particular set of parameter values to demonstrate that a fully Bayesian approach can capture a variety of aspects of participants' data, especially fixation patterns which have been largely overlooked in previous research. The parameters used in the simulation are σ_0_ = 4, δ = 0.005, γ = 0.1, λ = 1.1, κ = 2, ω = 2.5, and ω_0_ = −6.5 (see Table 1 for a summary description of model parameters). However, it is worth noting that we did examine our model over a large grid on the parameter space (Table 1). Our model simulation results did not strongly depend on the particular values of the parameters, and the behavior and fixation patterns in the Results section can be reproduced by a large proportion of parameters on the grid space.

**Table 1:** Model parameters and their range tested in the simulation.

**Parameter**	**Description**	**Parameter range tested in simulation**
σ_0_	The standard deviation of the sampling distributions.	[4 to 10]
δ	The decreasing step of decision threshold.	[0.0025 to 0.02]
γ	The discounting factor on the mean of the sampling distribution for the non-fixated item.	[0 to 0.9]
κ	The factor by which the sampling distribution of the non-fixated item is noisier than that of the fixated item.	[2 to 4]
λ	The expanding factor of belief uncertainty of the non-fixated item.	[1 to 1.25]
ω, ω_0_	The slope (sensitivity to uncertainty ratio) and intercept (saccade cost) of the parameters in the softmax decision function.	ω: [1 to 4] ω_0_: [−8 to −2]

## Results

### Choice Patterns and Fixation Biases

**Figure 2:**
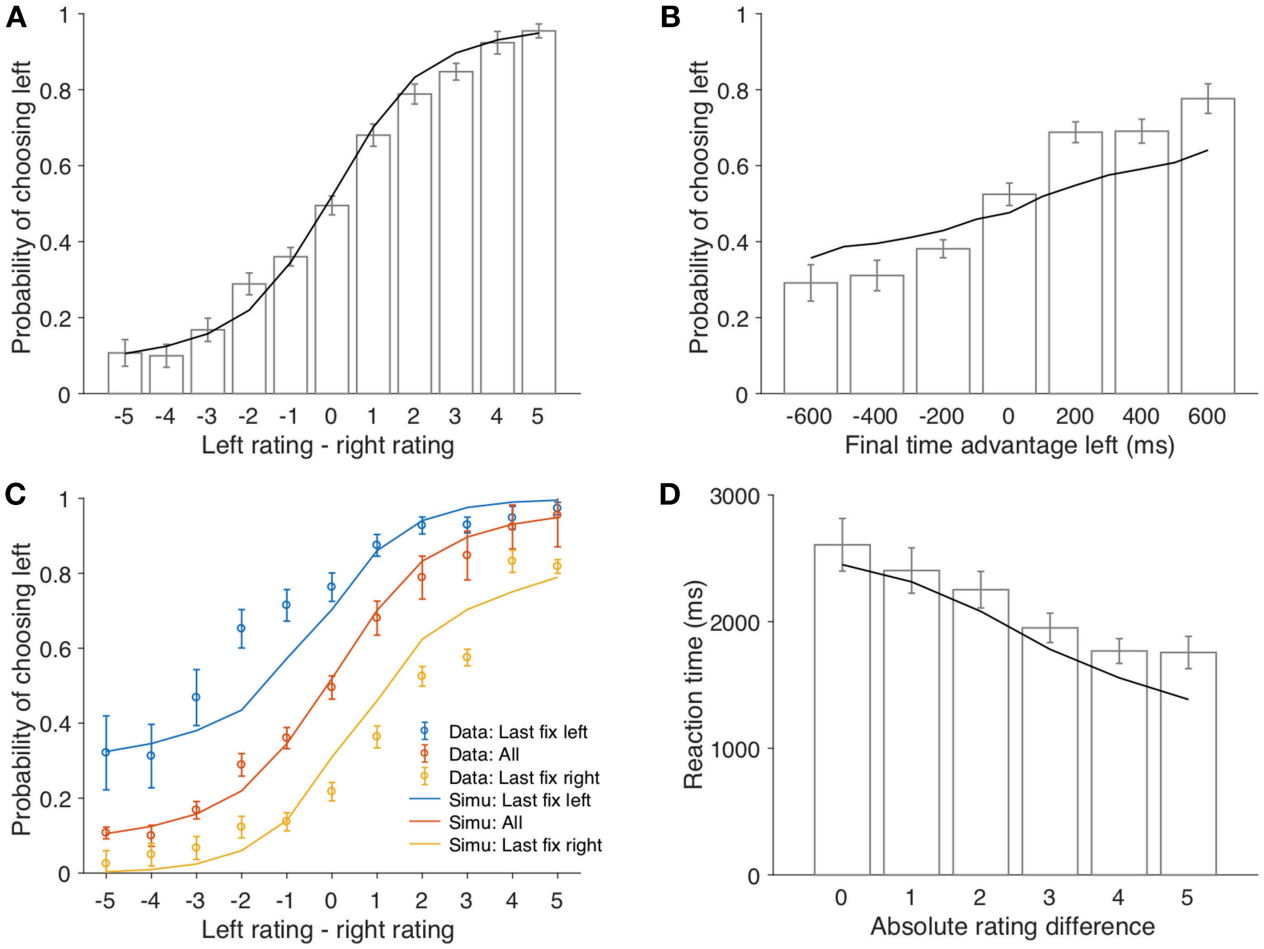
The model predictions of the behavioral patterns. **(A)** the probability of choosing the left item as a function of the rating difference between the two items (left-right). Bars represent the experimental data (error bars represent 1 s.e.m across all participants); the black line represents the model simulation results; same for **(B,D)**. **(B)** probability that the left item is chosen as a function of its total fixation duration advantage over the right item. **(C)** probability that the left item is chosen as a function of its rating advantage over the right item, conditioned on the last fixation. Yellow circles and the yellow line correspond to trials that participants looked at the left item in the last fixation; blue circles and the blue line correspond to trials that participants looked at the right item in the last fixation; red circles and the red line indicate the average of both. **(D)** reaction time as a function of absolute rating difference.

We first show that our model accounts for the core model predictions in the original paper (Krajbich et al., 2010). In the decision phase, participants' choices were consistent with how they rated the items in the rating phase: the higher they rated an item compared to its alternative, the more likely the item would be chosen (mixed effect logistic regression slope β = 0.60, *p* < 0.001; bars in Figure 2A). In our model, the value estimate for each item is obtained from sampling the underlying option value distribution, and this is reflected in the final choice (line in Figure 2A), consistent with previous literature that suggested choice predictions from aDDM can be incorporated in the Bayesian framework (Cassey et al., 2013). This is similar to the aDDM, where RDV was accumulated according to the (relative) difference between the values of two items. Participants were more likely to choose an item if they spent longer time looking at it (β = 0.0017, *p* < 0.001; Figure 2B) and if they were looking at it right before the choice selection (β = 0.61, *p* < 0.001; Figure 2C). Both effects were predicted by aDDM via the assumption that the drift rate of non-fixated item is discounted. Similarly, our model can explain both phenomena because it assumes value discounting for the non-fixated item (Equation 2): a longer fixation indicates a stronger discounting effect on the non-fixated item, resulting in a lower evaluation for the non-fixated item and thus a higher probability of choosing the fixated one. Similarly, provided everything else being equal, the item of last fixation enjoys more unbiased (undiscounted) evaluations before the choice, hence the higher chosen rate. It was also observed in the data that reaction time decreased as the absolute rating difference increased (β = −191.6, *p* < 0.001; Figure 2D). Evidence accumulation models such as aDDM explain this as it takes longer time to reach a decision threshold when the drift rate is smaller. Similar to aDDM (and as we noted previously, there is a fundamental equivalence of Bayesian approach and DDM Bitzer et al., 2014), our model interprets such behavioral pattern as fewer samples are needed to separate the two value distributions if the distance between them is larger.

### Eye-Movement Patterns

Standard DDM approaches usually are agnostic about participants' eye fixation patterns (but see Towal et al., 2013). For example, aDDM (Krajbich et al., 2010) sidestepped the mechanism of saccade and instead used the empirical fixation duration distribution as an input to the model to predict choice behavior. Although standard DDMs predict the distribution of total reaction time to be an inverse Gaussian distribution (note that Krajbich et al., 2010 used the log-normal distribution to capture their empirical saccade fixation data, probably due to the time-invariant noise term in the aDDM), they remain agnostic about the distribution of individual fixation duration (but see Towal et al., 2013). In contrast, our model speaks directly to participants' saccade patterns as they are the intermediate products between visual option inputs and the final behavioral choices. Indeed, these data provide a test bed for our framework and future efforts that explicitly model the eye-movement patterns.

**Figure 3:**
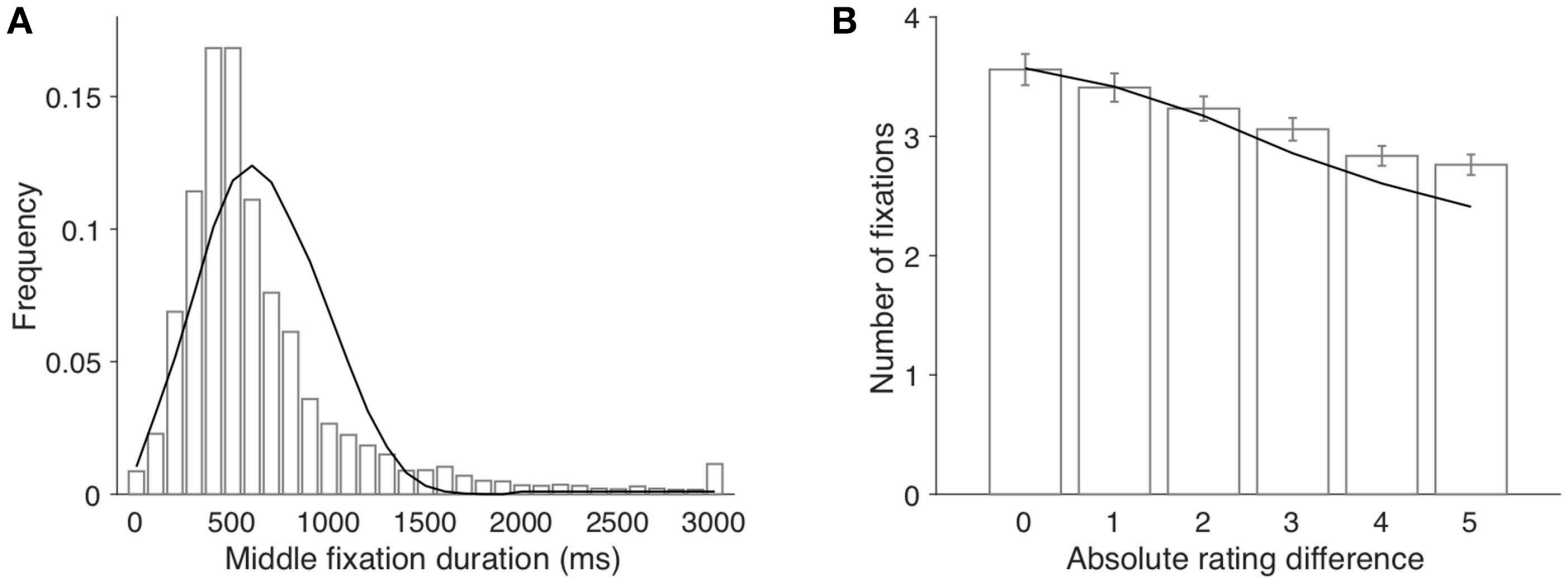
**(A)** The histogram of middle fixation duration and the model fit. Bars and line represent the empirical distribution and the simulated distribution, respectively. The last bin contains all fixations longer than 3,000 ms. **(B)** average number of fixations per trial as a function of absolute rating difference. Bars represent the empirical data (error bars indicate 1 s.e.m. across participants); the line represents model simulation results.

As shown in Figure 3A, the overall distribution of the middle fixation duration is skewed toward right, which is qualitatively captured by our simulation results. One interesting finding in Krajbich et al. (2010) was that the fixation number increased as the choice became more difficult, that is, when the absolute value difference was smaller (β = −0.16, *p* < 0.001; Figure 3B). The aDDM model in Krajbich et al. (2010) sidestepped this by sampling fixation durations from separate empirical distributions conditioned on absolute rating difference. In contrast, our model provides an intuitive and natural explanation for this effect: as the task gets more difficult (ratings of two items are closer), more samples are needed to separate the two underlying distributions. However, as more samples are taken from the fixated item, estimated uncertainty of the non-fixated item increases due to the forgetting effect. In order to make the choice with sufficient confidence, participants need to switch fixations between two items and evaluate them alternatively more often, resulting in more fixations in total. In brief, our model predicts that decision time and the number of fixations are intricately linked together. Indeed, our model simulation confirms this intuition: the simulation data predict the inverse relationship between the number of fixations and absolute value difference (line in Figure 3B).

**Figure 4:**
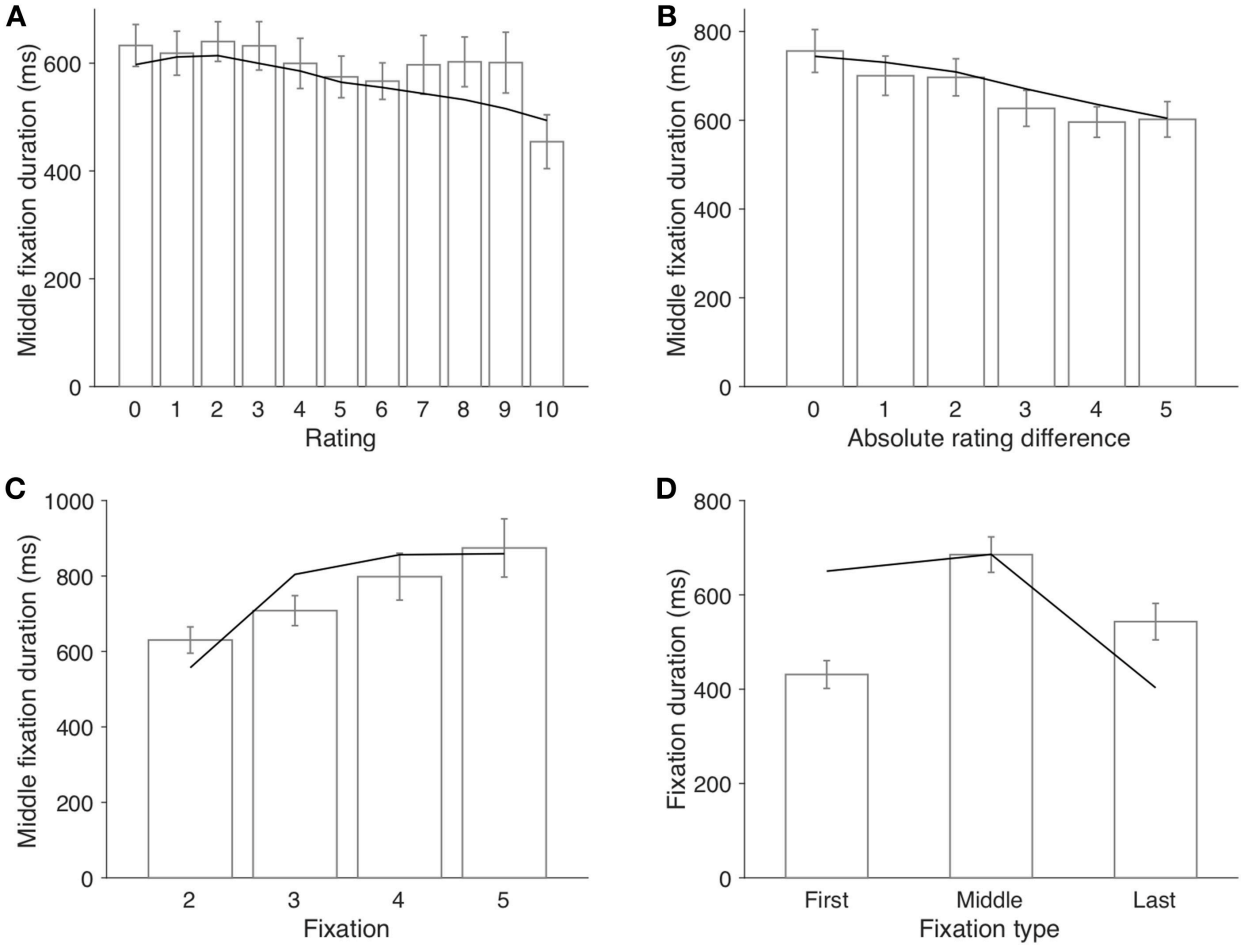
Factors that influence fixation duration. **(A)** middle fixation duration as a function of the item rating. **(B)** middle fixation duration as a function of absolute rating difference between two items. **(C)** middle fixation duration as a function of the index of fixation (trials with only one fixation are excluded from this analysis). **(D)** fixation duration by type. Middle fixations indicate the fixations that are not the first nor the last fixation in a trial. Bars represent the empirical data (error bars indicate 1 s.e.m. across participants); lines represent the model simulation results.

Another finding that supports a proactive sampling model is the fact that middle fixation duration was not correlated with item value itself (β = −5.91, *p* = 0.15; Figure 4A), but negatively correlated with absolute rating difference (β = −32.3, *p* < 0.001; Figure 4B). Again, aDDM took this pattern as given and used fixation durations directly sampled from the empirical distribution conditioned on absolute rating difference (Krajbich et al., 2010). In contrast, in our model, fixation switch is determined by the comparison of the uncertainties of value estimate of the two items, not the values themselves, so fixation duration does not vary as a function of individual item ratings (line in Figure 4A). When the rating difference between two items is large, the decision threshold is easy to surpass, even with a small number of samples. As a result, it is easier for a long fixation (consisting of many samples) to lead to a final choice and therefore become the final fixation. Thus, larger rating difference corresponds to shorter middle fixation duration on average (line in Figure 4B).

Another worth-noting pattern about middle fixation duration is that it increased steadily throughout a trial (β = 58.8, *p* = 0.0018; Figure 4C). Since 96.9% of trials terminated within six fixations, we focus on only the second to the fifth fixations (excluding the first and last fixations). Our model is constructed such that the fixation switching probability depends on the ratio of uncertainties of the two value estimation distributions. Toward the end of a trial, changes in the uncertainty ratio tends to decrease, rendering lower likelihood of fixation switch and thus longer fixations in later part of the trial (line in Figure 4C). It was observed in Krajbich et al. (2010) that the first fixation of a trial was shorter than middle fixations [paired *t* test, *t*_(38)_ = −9.33, *p* < 0.001; Figure 4D] and they set up two separate empirical distributions from which the model sampled first and middle fixations respectively. Our model predicts this pattern (line in Figure 4D) and sees it as a special case of the fact that fixation duration increases within a trial. The reversal pattern of the last fixation duration, however, is due to the “truncated” or premature middle fixations: decision process terminates when the threshold is reached despite whether current fixation would have continued otherwise, which makes this final fixation shorter than it could have been. Both aDDM and our model predict this phenomenon.

### Relationship Between **κ** and **λ**

**Figure 5:**
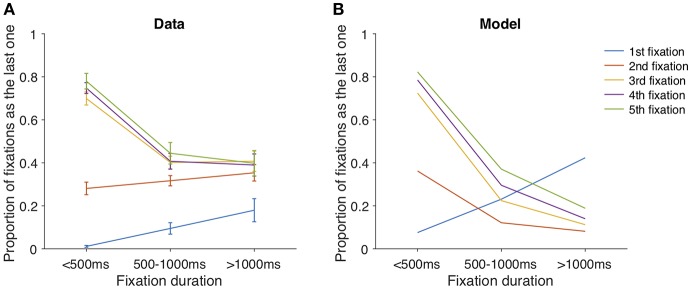
The proportion of fixations being the last of a trial, as the function of fixation duration and the index of fixation. **(A)** data. **(B)** model simulation.

It might seem arbitrary to introduce both the noise ratio κ and variance expansion factor λ in our model. At first glance, both parameters can lead to the seemingly equivalent “expanding” effect on uncertainty over the non-fixated item. However, even though the noise ratio κ leads to a noisier sampling distribution of the non-fixated item compared to the fixated one, getting new samples still helps making value estimate more accurate over time; in contrast, the variance expansion factor λ makes value estimate more uncertain over time. A closer examination of the fixation data reveals the necessity of both parameters: the relationship between the probability of committing a choice and the duration of fixation was modulated by the number of fixations (Figure 5A). In a model without the variance expansion effect (λ = 1), the participant will be more likely to commit a choice when spending more time sampling from items. However, the probability of making an explicit choice decreased as the fixation duration increased when fixation number is big (>2). The introduction of λ, due to its exponential form, creates the competition between the exponentially expanding (expansion) and hyperbolic updating (contraction) of non-fixated item variance. An interesting derivation of this antagonism is that the competition results depend on the fixation number because of the different forms of expansion and contraction functions. Indeed, our model simulation captures this dependence (Figure 5B), providing additional evidence that variance expansion, or the forgetting process is necessary to explain the fixation data. Additionally, when κ = 1, the uncertainty of both items will be the same throughout a trial, leading to a constant uncertainty ratio and hence stable switch probability. If that is the case, the middle fixation duration will be approximately the same throughout a trial. However, as shown in Figure 4C, the middle fixation duration increased as the trial proceeded, providing extra evidence for the necessity of the noise ratio parameter κ.

## Discussion

The evidence accumulation model has witnessed its great success in the past decades to account for the choice and reaction time data in the field of perceptual decision making (Ratcliff, 1978; Bogacz et al., 2006; Bogacz, 2007; Gold and Shadlen, 2007). In a typical experimental setup where stimuli (e.g., randomly moving dots) are presented together, the model predicts that participants appraise stimuli passively until the evidence accumulation of certain decision variable reaches a (predefined) threshold to engender an explicit choice. Later studies using electrophysiology mapped the integration function to neural activities in brain areas such as lateral intraparietal cortex (LIP) and frontal eye field (FEF) (Platt and Glimcher, 1999; Ditterich et al., 2003; Gold and Shadlen, 2007). Recently, such a theoretical approach has been adopted to study value-based decision where the typical setup involves options displayed at different locations of the visual field and eye movement data were also recorded (Armel et al., 2008; Krajbich et al., 2010, 2012; Krajbich and Rangel, 2011; Cassey et al., 2013; Towal et al., 2013). In addition to speed and accuracy data, the newly acquired saccade information provides a novel venue to understand the underlying decision-making mechanism. Indeed, it has been proposed that the choice preference can be driven by the fixation duration on certain option due to the asymmetric evidence accumulation between fixated and non-fixated options, probably caused by attentional bias (Krajbich et al., 2010, 2012; Krajbich and Rangel, 2011; Towal et al., 2013; Tavares et al., 2017). Furthermore, the disruption of such fixation leads to biased moral and value decisions (Armel et al., 2008; Krajbich et al., 2010; Pärnamets et al., 2015). However, the eye-tracking data also pose a theoretical challenge: what drives the eye fixation in such tasks? Inspired by the optimal sampling theory, in this work, we presented a Bayesian generative model for eye-movement in a value-based binary choice task (Yuille and Bülthoff, 1996; Summerfield and Tsetsos, 2012; Bitzer et al., 2014). The model fits well to the participants' choices, as well as the choice biases induced by fixation and the effect of decision difficulty. More importantly, it makes novel and testable predictions of the fixation duration distribution and fixation patterns as functions of option attractiveness ratings and the index of fixation, some of which were reported in Krajbich et al. (2010) and others are newly identified in the current work.

Eye movement has been reported to be causally linked with valuation and choice generation in value-based decision making (Armel et al., 2008; Krajbich et al., 2010; Krajbich and Rangel, 2011), but formal theories explaining why and how people make eye movements during such decisions are lacking. The fact that our model is capable of explaining reaction time, choice and eye fixation data indicates that people might not passively accumulate value or perceptual information as standard DDM suggests; instead, they actively switch their fixations to maximize information gain before committing to a choice decision. Similar concepts such as Infomax algorithm have been introduced before in perception decision making and the research area of artificial intelligence (Butko and Movellan, 2010).

For simplicity, we omitted physiological details that might constrain the physical speed of information processing and eye repositioning cost. For example, it has been reported that the activity delay between retina and the FEF in awake monkey is 75 ± 10 ms (assuming a Gaussian distribution), FEF and saccade 30 ± 10 ms, and LIP and saccade 90 ± 10 ms (Wurtz and Goldberg, 1972; Schmolesky et al., 1998; Towal et al., 2013). Instead, we assume a rather crude individual uniform sampling interval between 50 and 150 ms, and set repositioning cost as a free parameter in the logistic function (as behavioral costs in Ahmad et al., 2014). Surprisingly, our model is able to capture various aspects of participants' data despite the simplified assumption above, proving the robustness of such a quantitative approach. Of note is the discrepancy between our model predictions and fixation data in Figure 5, where the model overestimates the probability that a trial terminates over only one fixation (blue curves). The first fixation is unique since our model assumes that participants are able to sample from both options, irrespective of current fixation location, potentially due to rumination and endogenous attention. However, during first fixation, it is impossible for participants to ruminate on an option they have not yet observed. So, it is plausible that the cognitive mechanism of sampling from the non-fixated item can be inherently different during the first fixation (before the participant has the chance to look at the alternative item for the first time), compared to later fixations. We decide to keep the model simple and concise such that it can be generalized to other decision contexts.

A few recent studies also examined the eye fixation pattern in value-based decisions (Cassey et al., 2013; Towal et al., 2013). For example, Towal et al. (2013) suggested that the combination of visual salience and value of different options drives the fixation switch, which further helps shape participant's actual choice. Item values are therefore used twice in predicting choice. This view is in contrast with earlier research that advocated the reverse causality between fixation duration and value difference (Krajbich et al., 2010). Our model challenges this view and instead proposes that eye fixation switch acts as an active information gathering process by comparing the levels of uncertainties between two estimated value distributions.

The newly added dimension of fixation pattern data, in addition to the traditional speed and accuracy information in perceptual and economic decision-making tasks, has provided an exciting testbed for candidate decision theories that emphasize the interplay between eye-movement and choice selection. Our model is among the first to provide a unified framework to account for different levels of complexities in the fixation pattern data and can be easily extended to multiple option paradigms.

## Data Availability Statement

The data analyzed in this study was obtained from Drs. Ian Krajbich, Carrie Armel, and Antonio Rangel. Requests to access these datasets should be directed to these authors.

## Author Contributions

MS, XW, HZ, and JL conceived the concept. MS and XW performed the analysis. MS, XW, HZ, and JL wrote the manuscript.

### Conflict of Interest Statement

The authors declare that the research was conducted in the absence of any commercial or financial relationships that could be construed as a potential conflict of interest.
